# Supplementary material for: Associations between plasma proteins and psychological wellbeing: evidence from over 20 years of the English Longitudinal Study of Ageing
Source: medRxiv. 2025 Sep 30:2025.02.05.25321715. Originally published 2025 Feb 6. Preprint. [Version 3] doi: 10.1101/2025.02.05.25321715 (PMC11838684; doi:10.1101/2025.02.05.25321715)
Supplement: Supplementary file 1 [file media-1.docx]

**Table 1. Baseline characteristics of the ELSA participants at study wave 4.**

| **Characteristics** | **N=3,262** |
| --- | --- |
| Age, Mean (SD) | 63.5 (9.4) |
| *Missing, N (%)* | *1 (0.03)* |
| Female, N (%) | 1,793 (55.0) |
| White ethnicity, N (%) | 3,163 (97.0) |
| Non-pension wealth quintile, N (%) |  |
| 1st Poorest | 592 (18.6) |
| 2nd | 625 (19.6) |
| 3rd | 625 (19.6) |
| 4th | 618 (19.4) |
| 5th Richest | 729 (22.9) |
| *Missing, N (%)* | *73 (2.2)* |
| Smoking status, N (%) |  |
| Never smoker | 1323 (40.6) |
| Former smoker | 1480 (45.4) |
| Current smoker | 452 (13.9) |
| *Missing, N (%)* | *7 (0.2)* |
| Body mass index, Mean (SD) | 28.1 (5.1) |
| *Missing, N (%)* | *70 (2.1)* |
| Cardiovascular disease, N (%) | 169 (5.2) |
| Stroke, N (%) | 51 (1.6) |
| Cancer, N (%) | 56 (1.7) |
| Diabetes, N (%) | 150 (4.6) |
| Arthritis, N (%) | 632 (19.4) |
| Chronic lung disease, N (%) | 66 (2.0) |
| **Wave 4 Wellbeing indicators** |  |
| CASP score - hedonic, Mean (SD) [ranged 0 – 12] | 10.0 (1.8) |
| *Missing, N (%)* | *386 (11.8)* |
| CASP score - eudaimonic, Mean (SD) [ranged 0 – 42] | 28.6 (6.9) |
| *Missing, N (%)* | *461 (14.1)* |
| SWLS score, Mean (SD) [ranged 0 – 30] | 20.4 (6.2) |
| *Missing, N (%)* | *435 (13.3)* |
| CESD score, Mean (SD) [ranged 0 – 8] | 1.3 (1.8) |
| *Missing, N (%)* | *19 (0.58)* |
| Depression (CESD ≥ 3), N (%) | 592 (18.1) |
| *Missing, N (%)* | *19 (0.58)* |

SD, standard deviation; CASP, Control, Autonomy, Self-Realization and Pleasure; SWLS, Diener Life Satisfaction scale; CESD, Center for Epidemiological Studies-Depression scale.
