# Supplementary material for: Associations between plasma proteins and psychological wellbeing: evidence from over 20 years of the English Longitudinal Study of Ageing
Source: medRxiv. 2025 Apr 7:2025.02.05.25321715. Originally published 2025 Feb 6. Preprint. [Version 2] doi: 10.1101/2025.02.05.25321715 (PMC11838684; doi:10.1101/2025.02.05.25321715)
Supplement: Supplementary file 1 [file media-1.docx]

**Supplementary materials**

**Supplementary Table 1. Full list of Olink proteins assayed in ELSA.**

| **Uniprot ID** | **Gene** | **Protein name** |
| --- | --- | --- |
| Q9BTE6 | AARSD1 | Alanyl-tRNA editing protein Aarsd1 |
| Q96IU4 | ABHD14B | Protein ABHD14B |
| Q9BYF1 | ACE2 | Angiotensin-converting enzyme 2 |
| P37023 | ACVRL1 | Serine/threonine-protein kinase receptor R3 |
| Q13444 | ADAM15 | Disintegrin and metalloproteinase domain-containing protein 15 |
| Q9P0K1 | ADAM22 | Disintegrin and metalloproteinase domain-containing protein 22 |
| O75077 | ADAM23 | Disintegrin and metalloproteinase domain-containing protein 23 |
| Q76LX8 | ADAMTS13 | A disintegrin and metalloproteinase with thrombospondin motifs 13 |
| O60242 | ADGRB3 | Adhesion G protein-coupled receptor B3 |
| P35318 | ADM | Pro-adrenomedullin |
| Q15109 | AGER | Advanced glycosylation end product-specific receptor |
| O00253 | AGRP | Agouti-related protein |
| Q96B36 | AKT1S1 | Proline-rich AKT1 substrate 1 |
| P02760 | AMBP | Protein AMBP |
| Q15389 | ANGPT1 | Angiopoietin-1 |
| Q9UJ72 | ANXA10 | Annexin A10 |
| P19801 | AOC1 | Amiloride-sensitive amine oxidase [copper-containing] |
| Q9NR71 | ASAH2 | Neutral ceramidase |
| P07306 | ASGR1 | Asialoglycoprotein receptor 1 |
| Q16864 | ATP6V1F | V-type proton ATPase subunit F |
| Q96GW7 | BCAN | Brevican core protein |
| P12644 | BMP4 | Bone morphogenetic protein 4 |
| P22004 | BMP6 | Bone morphogenetic protein 6 |
| Q9BWV1 | BOC | Brother of CDO |
| Q10589 | BST2 | Bone marrow stromal antigen 2 |
| P35218 | CA5A | Carbonic anhydrase 5A |
| Q8N126 | CADM3 | Cell adhesion molecule 3 |
| Q9Y2V2 | CARHSP1 | Calcium-regulated heat-stable protein 1 |
| P27352 | CBLIF | Cobalamin binding intrinsic factor |
| Q92583 | CCL17 | C-C motif chemokine 17 |
| Q9Y4X3 | CCL27 | C-C motif chemokine 27 |
| P10147 | CCL3 | C-C motif chemokine 3 |
| P41217 | CD200 | OX-2 membrane glycoprotein |
| Q8TD46 | CD200R1 | Cell surface glycoprotein CD200 receptor 1 |
| Q08708 | CD300C | CMRF35-like molecule 6 |
| Q8TDQ1 | CD300LF | CMRF35-like molecule 1 |
| Q8IX05 | CD302 | CD302 antigen |
| P20138 | CD33 | Myeloid cell surface antigen CD33 |
| P28907 | CD38 | ADP-ribosyl cyclase/cyclic ADP-ribose hydrolase 1 |
| P01730 | CD4 | T-cell surface glycoprotein CD4 |
| P29965 | CD40LG | CD40 ligand |
| P08962 | CD63 | CD63 antigen |
| Q9UIB8 | CD84 | SLAM family member 5 |
| P55291 | CDH15 | Cadherin-15 |
| Q12864 | CDH17 | Cadherin-17 |
| P22223 | CDH3 | Cadherin-3 |
| P55285 | CDH6 | Cadherin-6 |
| P40198 | CEACAM3 | Carcinoembryonic antigen-related cell adhesion molecule 3 |
| P31997 | CEACAM8 | Carcinoembryonic antigen-related cell adhesion molecule 8 |
| Q9Y5P4 | CERT1 | Ceramide transfer protein |
| P41208 | CETN2 | Centrin-2 |
| Q8IUN9 | CLEC10A | C-type lectin domain family 10 member A |
| Q9P126 | CLEC1B | C-type lectin domain family 1 member B |
| O94985 | CLSTN1 | Calsyntenin-1 |
| O94779 | CNTN5 | Contactin-5 |
| P48052 | CPA2 | Carboxypeptidase A2 |
| P14384 | CPM | Carboxypeptidase M |
| P78560 | CRADD | Death domain-containing protein CRADD |
| P52943 | CRIP2 | Cysteine-rich protein 2 |
| O95727 | CRTAM | Cytotoxic and regulatory T-cell molecule |
| P15509 | CSF2RA | Granulocyte-macrophage colony-stimulating factor receptor subunit alpha |
| P09919 | CSF3 | Granulocyte colony-stimulating factor |
| Q16619 | CTF1 | Cardiotrophin-1 |
| Q99895 | CTRC | Chymotrypsin-C |
| P53634 | CTSC | Dipeptidyl peptidase 1 |
| P07711 | CTSL | Cathepsin L1 |
| P25774 | CTSS | Cathepsin S |
| P09341 | CXCL1 | Growth-regulated alpha protein |
| P07585 | DCN | Decorin |
| Q08345 | DDR1 | Epithelial discoidin domain-containing receptor 1 |
| Q16698 | DECR1 |  |
| O15263 | DEFB4A_DEFB4B | Beta-defensin 4A |
| O94907 | DKK1 | Dickkopf-related protein 1 |
| Q9UBT3 | DKK4 | Dickkopf-related protein 4 |
| P16444 | DPEP1 | Dipeptidase 1 |
| Q9H4A9 | DPEP2 | Dipeptidase 2 |
| Q8NBI3 | DRAXIN | Draxin |
| P32926 | DSG3 | Desmoglein-3 |
| P51452 | DUSP3 | Dual specificity protein phosphatase 3 |
| Q14213_Q8NEV9 | EBI3_IL27 | Interleukin-27 |
| P42892 | ECE1 | Endothelin-converting enzyme 1 |
| Q9HAV5 | EDA2R | Tumor necrosis factor receptor superfamily member 27 |
| P52798 | EFNA4 | Ephrin-A4 |
| P23588 | EIF4B | Eukaryotic translation initiation factor 4B |
| Q5JZY3 | EPHA10 | Ephrin type-A receptor 10 |
| O15197 | EPHB6 | Ephrin type-B receptor 6 |
| O14944 | EREG | Proepiregulin |
| P15311 | EZR | Ezrin |
| P25116 | F2R | Proteinase-activated receptor 1 |
| P13726 | F3 | Tissue factor |
| P12104 | FABP2 | Fatty acid-binding protein |
| P51161 | FABP6 | Gastrotropin |
| P24071 | FCAR | Immunoglobulin alpha Fc receptor |
| P31994 | FCGR2B | Low affinity immunoglobulin gamma Fc region receptor II-b |
| Q96LA5 | FCRL2 | Fc receptor-like protein 2 |
| Q9NSA1 | FGF21 | Fibroblast growth factor 21 |
| Q9GZV9 | FGF23 | Fibroblast growth factor 23 |
| P21802 | FGFR2 | Fibroblast growth factor receptor 2 |
| P49789 | FHIT | Bis(5'-adenosyl)-triphosphatase |
| Q13451 | FKBP5 | Peptidyl-prolyl cis-trans isomerase FKBP5 |
| Q9Y680 | FKBP7 | Peptidyl-prolyl cis-trans isomerase FKBP7 |
| O43155 | FLRT2 | Leucine-rich repeat transmembrane protein FLRT2 |
| Q92765 | FRZB | Secreted frizzled-related protein 3 |
| P19883 | FST | Follistatin |
| Q9BYC5 | FUT8 | Alpha-(1 |
| P32456 | GBP2 | Guanylate-binding protein 2 |
| Q9UK05 | GDF2 | Growth/differentiation factor 2 |
| P39905 | GDNF | Glial cell line-derived neurotrophic factor |
| P56159 | GFRA1 | GDNF family receptor alpha-1 |
| O60609 | GFRA3 | GDNF family receptor alpha-3 |
| P36269 | GGT5 | Glutathione hydrolase 5 proenzyme |
| P01241 | GH1 | Somatotropin |
| Q04760 | GLO1 | Lactoylglutathione lyase |
| P78333 | GPC5 | Glypican-5 |
| Q14956 | GPNMB | Transmembrane glycoprotein NMB |
| P09211 | GSTP1 | Glutathione S-transferase P |
| P12544 | GZMA | Granzyme A |
| Q16775 | HAGH | Hydroxyacylglutathione hydrolase |
| Q9UJM8 | HAO1 | Hydroxyacid oxidase 1 |
| Q96D42 | HAVCR1 | Hepatitis A virus cellular receptor 1 |
| Q99075 | HBEGF | Proheparin-binding EGF-like growth factor |
| P09601 | HMOX1 | Heme oxygenase 1 |
| P30519 | HMOX2 | Heme oxygenase 2 |
| P14625 | HSP90B1 | Endoplasmin |
| P04792 | HSPB1 | Heat shock protein beta-1 |
| P35475 | IDUA | Alpha-L-iduronidase |
| P13284 | IFI30 | Gamma-interferon-inducible lysosomal thiol reductase |
| Q8IU54 | IFNL1 | Interferon lambda-1 |
| Q9Y6K9 | IKBKG | NF-kappa-B essential modulator |
| Q9UKS7 | IKZF2 | Zinc finger protein Helios |
| P29459_P29460 | IL12A_IL12B | Interleukin-12 |
| P40933 | IL15 | Interleukin-15 |
| Q14005 | IL16 | Pro-interleukin-16 |
| Q8TAD2 | IL17D | Interleukin-17D |
| Q14116 | IL18 | Interleukin-18 |
| Q9HB29 | IL1RL2 | Interleukin-1 receptor-like 2 |
| P18510 | IL1RN | Interleukin-1 receptor antagonist protein |
| P24001 | IL32 | Interleukin-32 |
| P26951 | IL3RA | Interleukin-3 receptor subunit alpha |
| P24394 | IL4R | Interleukin-4 receptor subunit alpha |
| Q01344 | IL5RA | Interleukin-5 receptor subunit alpha |
| P05231 | IL6 | Interleukin-6 |
| Q9H0C8 | ILKAP | Integrin-linked kinase-associated serine/threonine phosphatase 2C |
| P29218 | IMPA1 | Inositol monophosphatase 1 |
| Q9UK53 | ING1 | Inhibitor of growth protein 1 |
| Q6UXK2 | ISLR2 | Immunoglobulin superfamily containing leucine-rich repeat protein 2 |
| Q9UKP3 | ITGB1BP2 | Integrin beta-1-binding protein 2 |
| P57087 | JAM2 | Junctional adhesion molecule B |
| Q96EK5 | KIFBP | KIF-binding protein |
| P43628 | KIR2DL3 | Killer cell immunoglobulin-like receptor 2DL3 |
| Q6UWL6 | KIRREL2 | Kin of IRRE-like protein 2 |
| P21583 | KITLG | Kit ligand |
| Q86Z14 | KLB | Beta-klotho |
| Q16719 | KYNU | Kynureninase |
| Q6ISS4 | LAIR2 | Leukocyte-associated immunoglobulin-like receptor 2 |
| O43561 | LAT | Linker for activation of T-cells family member 1 |
| Q6UX15 | LAYN | Layilin |
| P41159 | LEP | Leptin |
| P48357 | LEPR | Leptin receptor |
| O00214 | LGALS8 | Galectin-8 |
| O00182 | LGALS9 | Galectin-9 |
| P06858 | LPL | Lipoprotein lipase |
| P30533 | LRPAP1 | Alpha-2-macroglobulin receptor-associated protein |
| Q9NS15 | LTBP3 | Latent-transforming growth factor beta-binding protein 3 |
| Q9BS40 | LXN | Latexin |
| Q9Y6D9 | MAD1L1 | Mitotic spindle assembly checkpoint protein MAD1 |
| P55145 | MANF | Mesencephalic astrocyte-derived neurotrophic factor |
| P10636 | MAPT | Microtubule-associated protein tau |
| Q9UEW3 | MARCO | Macrophage receptor MARCO |
| O15232 | MATN3 | Matrilin-3 |
| Q8NFP4 | MDGA1 | MAM domain-containing glycosylphosphatidylinositol anchor protein 1 |
| Q12866 | MERTK | Tyrosine-protein kinase Mer |
| P08473 | MME | Neprilysin |
| P39900 | MMP12 | Macrophage metalloelastase |
| P09237 | MMP7 | Matrilysin |
| P21757 | MSR1 | Macrophage scavenger receptor types I and II |
| O14793 | MSTN | Growth/differentiation factor 8 |
| P41227 | NAA10 | N-alpha-acetyltransferase 10 |
| Q02083 | NAAA | N-acylethanolamine-hydrolyzing acid amidase |
| P41271 | NBL1 | Neuroblastoma suppressor of tumorigenicity 1 |
| O14594 | NCAN | Neurocan core protein |
| Q92597 | NDRG1 | Protein NDRG1 |
| P07196 | NEFL | Neurofilament light polypeptide |
| P01138 | NGF | Beta-nerve growth factor |
| Q9HAN9 | NMNAT1 | Nicotinamide/nicotinic acid mononucleotide adenylyltransferase 1 |
| P06748 | NPM1 | Nucleophosmin |
| P16860 | NPPB | Natriuretic peptides B |
| Q92823 | NRCAM | Neuronal cell adhesion molecule |
| O60462 | NRP2 | Neuropilin-2 |
| Q16620 | NTRK2 | BDNF/NT-3 growth factors receptor |
| Q16288 | NTRK3 | NT-3 growth factor receptor |
| P58417 | NXPH1 | Neurexophilin-1 |
| P78380 | OLR1 | Oxidized low-density lipoprotein receptor 1 |
| Q8IYS5 | OSCAR | Osteoclast-associated immunoglobulin-like receptor |
| P09466 | PAEP | Glycodelin |
| Q13219 | PAPPA | Pappalysin-1 |
| P09874 | PARP1 | Poly [ADP-ribose] polymerase 1 |
| Q9BQ51 | PDCD1LG2 | Programmed cell death 1 ligand 2 |
| P01127 | PDGFB | Platelet-derived growth factor subunit B |
| P16234 | PDGFRA | Platelet-derived growth factor receptor alpha |
| Q9UHV9 | PFDN2 | Prefoldin subunit 2 |
| P49763 | PGF | Placenta growth factor |
| Q8TCT1 | PHOSPHO1 | Phosphoethanolamine/phosphocholine phosphatase |
| P01833 | PIGR | Polymeric immunoglobulin receptor |
| O15496 | PLA2G10 | Group 10 secretory phospholipase A2 |
| O43157 | PLXNB1 | Plexin-B1 |
| Q9ULL4 | PLXNB3 | Plexin-B3 |
| Q15126 | PMVK | Phosphomevalonate kinase |
| P63098 | PPP3R1 | Calcineurin subunit B type 1 |
| P51888 | PRELP | Prolargin |
| Q9BQR3 | PRSS27 | Serine protease 27 |
| Q16651 | PRSS8 | Prostasin |
| Q9NRG1 | PRTFDC1 | Phosphoribosyltransferase domain-containing protein 1 |
| Q2VWP7 | PRTG | Protogenin |
| P11464 | PSG1 | Pregnancy-specific beta-1-glycoprotein 1 |
| Q06323 | PSME1 | Proteasome activator complex subunit 1 |
| P18031 | PTPN1 | Tyrosine-protein phosphatase non-receptor type 1 |
| Q03393 | PTS | 6-pyruvoyl tetrahydrobiopterin synthase |
| P26022 | PTX3 | Pentraxin-related protein PTX3 |
| P15151 | PVR | Poliovirus receptor |
| Q9H477 | RBKS | Ribokinase |
| P00797 | REN | Renin |
| Q96B86 | RGMA | Repulsive guidance molecule A |
| Q6NW40 | RGMB | RGM domain family member B |
| Q96EP0 | RNF31 | E3 ubiquitin-protein ligase RNF31 |
| Q9HCK4 | ROBO2 | Roundabout homolog 2 |
| P23443 | RPS6KB1 | Ribosomal protein S6 kinase beta-1 |
| Q2MKA7 | RSPO1 | R-spondin-1 |
| Q6ZMJ2 | SCARA5 | Scavenger receptor class A member 5 |
| Q14108 | SCARB2 | Lysosome membrane protein 2 |
| Q96GP6 | SCARF2 | Scavenger receptor class F member 2 |
| Q14242 | SELPLG | P-selectin glycoprotein ligand 1 |
| Q8IW75 | SERPINA12 | Serpin A12 |
| Q8N474 | SFRP1 | Secreted frizzled-related protein 1 |
| Q9BZZ2 | SIGLEC1 | Sialoadhesin |
| Q9Y336 | SIGLEC9 | Sialic acid-binding Ig-like lectin 9 |
| Q9NQ25 | SLAMF7 | SLAM family member 7 |
| Q9H4F8 | SMOC1 | SPARC-related modular calcium-binding protein 1 |
| Q9H3U7 | SMOC2 | SPARC-related modular calcium-binding protein 2 |
| P17405 | SMPD1 | Sphingomyelin phosphodiesterase |
| O76070 | SNCG | Gamma-synuclein |
| P04179 | SOD2 | Superoxide dismutase [Mn] |
| Q99523 | SORT1 | Sortilin |
| Q08629 | SPOCK1 | Testican-1 |
| Q9BUD6 | SPON2 | Spondin-2 |
| P12931 | SRC | Proto-oncogene tyrosine-protein kinase Src |
| P37108 | SRP14 | Signal recognition particle 14 kDa protein |
| Q13043 | STK4 | Serine/threonine-protein kinase 4 |
| Q99426 | TBCB | Tubulin-folding cofactor B |
| P13385 | TDGF1 | Teratocarcinoma-derived growth factor 1 |
| Q02763 | TEK | Angiopoietin-1 receptor |
| P21980 | TGM2 | Protein-glutamine gamma-glutamyltransferase 2 |
| P07204 | THBD | Thrombomodulin |
| P35442 | THBS2 | Thrombospondin-2 |
| P40225 | THPO | Thrombopoietin |
| P04216 | THY1 | Thy-1 membrane glycoprotein |
| Q9H3S3 | TMPRSS5 | Transmembrane protease serine 5 |
| O00220 | TNFRSF10A | Tumor necrosis factor receptor superfamily member 10A |
| O14763 | TNFRSF10B | Tumor necrosis factor receptor superfamily member 10B |
| Q9Y6Q6 | TNFRSF11A | Tumor necrosis factor receptor superfamily member 11A |
| Q9NP84 | TNFRSF12A | Tumor necrosis factor receptor superfamily member 12A |
| O14836 | TNFRSF13B | Tumor necrosis factor receptor superfamily member 13B |
| Q96RJ3 | TNFRSF13C | Tumor necrosis factor receptor superfamily member 13C |
| O75509 | TNFRSF21 | Tumor necrosis factor receptor superfamily member 21 |
| Q92752 | TNR | Tenascin-R |
| Q9BW30 | TPPP3 | Tubulin polymerization-promoting protein family member 3 |
| Q969M7 | UBE2F | NEDD8-conjugating enzyme UBE2F |
| Q9BZM5 | ULBP2 | UL16-binding protein 2 |
| O95185 | UNC5C | Netrin receptor UNC5C |
| O43915 | VEGFD | Vascular endothelial growth factor D |
| Q96IQ7 | VSIG2 | V-set and immunoglobulin domain-containing protein 2 |
| Q6UX27 | VSTM1 | V-set and transmembrane domain-containing protein 1 |
| Q2TAL6 | VWC2 | Brorin |
| Q96NZ8 | WFIKKN1 | WAP |
| O00308 | WWP2 | NEDD4-like E3 ubiquitin-protein ligase WWP2 |
| P47992 | XCL1 | Lymphotactin |

**Supplementary Figure 1. Schematic diagram of participant selection in ELSA proteomics study.**

**Supplementary Figure 2. Correlation heatmap for eudaimonic wellbeing, hedonic wellbeing, life satisfaction and depressive symptoms by study wave.**

Life satisfaction was not measured in wave 1.

**Supplementary Figure 3. Volcano plots visualizing protein concentration associated with cross-sectional eudaimonic wellbeing, hedonic wellbeing, depressive symptoms, and life satisfaction at protein measurement from linear regressions, with minimal adjustments.**

Linear regression models for protein-wellbeing associations at wave 4. Models adjusted for age, sex, wealth quintile, ethnicity.

**Supplementary Figure 4. Volcano plots visualizing protein concentration associated with cross-sectional and longitudinal depression from fully adjusted logistic and mixed-effects logistic regressions.**

For cross-sectional associations, logistic regression models adjusted for age, sex, wealth quintile, ethnicity, smoking status, arthritis, cancer, stroke, chronic lung disease, diabetes, cardiovascular diseases.

For longitudinal associations, mixed effect logistic regression models allowed for protein interacting with time since protein measurements and age, and adjusted for age, sex, wealth quintile, ethnicity, smoking status, arthritis, cancer, stroke, chronic lung disease, diabetes, cardiovascular diseases.

**Supplementary Figure 5. Volcano plots visualizing protein concentration associated with cross-sectional eudaimonic wellbeing, hedonic wellbeing, depressive symptoms, and life satisfaction at protein measurement from linear regressions, with multiple adjustments.**

Linear regression models for protein-wellbeing associations at wave 4. Models adjusted for age, sex, wealth quintile, ethnicity, smoking status, arthritis, cancer, stroke, chronic lung disease, diabetes, cardiovascular diseases.

**Supplementary Figure 6. Volcano plots visualizing protein concentration associated with eudaimonic wellbeing, hedonic wellbeing, depressive symptoms, and life satisfaction after protein measurement from linear mixed effect regressions, with minimal adjustments.**

Linear mixed effect regression models for protein-wellbeing associations from wave 4 to wave 10. Models allowed for protein interacting with time since protein measurements and age, and adjusted for sex, wealth quintile, ethnicity.

**Supplementary Figure 7. Volcano plots visualizing protein concentration associated with eudaimonic wellbeing, hedonic wellbeing, depressive symptoms, and life satisfaction after protein measurement from linear mixed effect regressions, with multiple adjustments.**

Linear mixed effect regression models for protein-wellbeing associations from wave 4 to wave 10. Models allowed for protein interacting with time since protein measurements and age, and adjusted for age, sex, wealth quintile, ethnicity, smoking status, arthritis, cancer, stroke, chronic lung disease, diabetes, cardiovascular diseases, and body mass index.

**Supplementary Figure 8. Volcano plots visualizing protein concentration associated with eudaimonic wellbeing, hedonic wellbeing, depressive symptoms, and life satisfaction before protein measurement from linear mixed effect regressions, with minimal adjustments.**

Linear mixed effect regression models for protein-wellbeing associations from wave 1 to wave 4 (except for life satisfaction, while was from wave 2 to wave 4).  Models allowed for protein interacting with time since protein measurements and age, and adjusted for sex, wealth quintile, ethnicity.

**Supplementary Figure 9. Volcano plots visualizing protein concentration associated with eudaimonic wellbeing, hedonic wellbeing, depressive symptoms, and life satisfaction before protein measurement from linear mixed effect regressions, with multiple adjustments.**

Linear mixed effect regression models for protein-wellbeing associations from wave 1 to wave 4 (except for life satisfaction, while was from wave 2 to wave 4).  Models allowed for protein interacting with time since protein measurements and age, and adjusted for sex, wealth quintile, ethnicity, smoking status, arthritis, cancer, stroke, chronic lung disease, diabetes, cardiovascular diseases and body mass index.
